# Woven EndoBridge intrasaccular therapy for the treatment of unruptured wide-necked bifurcation aneurysms: a prospective study in a Chinese population

**DOI:** 10.1186/s41016-025-00418-2

**Published:** 2026-01-20

**Authors:** Chuan He, Jing Xu, Xu Gao, Guilin Li, Guobiao Liang, Yu Jun, Zhenwei Zhao, Bing Fang, Xiaodong Xie, Aihua Liu, Jianmin Zhang, Hongqi Zhang, Jianmin Liu

**Affiliations:** 1 Department of Neurosurgery, Xuanwu Hospital, Capital Medical University, Beijing, China; 2 China International Neuroscience Institute, Beijing, China; 3 Department of Neurosurgery, The Second Affiliated Hospital, Zhejiang University School of Medicine, Hangzhou, China; 4 Department of Neurosurgery, The General Hospital of Northern Theater Command of the Chinese People’s Liberation Army, Shenyang, China; 5 Department of Neurosurgery, Tangdu Hospital of Air Force Military Medical University, Xi’an, China; 6 Department of Neurosurgery, West China Hospital, Sichuan University, Chengdu, China; 7 Department of Interventional Neuroradiology, Beijing Tiantan Hospital, Capital Medical University, Beijing, China; 8 Neurovascular Center, Naval Medical University Changhai Hospital, Shanghai, China

**Keywords:** Aneurysm, WEB device, Endovascular, Wide-necked, Bifurcation, Complete occlusion

## Abstract

**Background:**

Woven EndoBridge (WEB™) has been shown to be safe and effective in the treatment of wide-necked bifurcation aneurysms (WNBAs). However, the use of this device has not been studied in China. This study assessed safety and effectiveness of WEB for the treatment of intracranial WNBAs in a Chinese population.

**Methods:**

The WEB Intrasaccular Therapy China Study (WEB-IT China) was a prospective, single-arm study allowing enrollment of adult WNBA patients treated with the WEB device between June 2017 and August 2019 among 8 centers in China. The primary effectiveness endpoint was treatment success rate, defined as complete aneurysm occlusion without retreatment, recurrent subarachnoid hemorrhage (SAH), or >50% parent artery stenosis at 1 year. The primary safety endpoint was the proportion of patients with major adverse event (MAE) incidence at 1 year follow-up, including non-accidental death or any major stroke within 30 days, or major ipsilateral stroke, or neurological death from day 31 to 1 year after treatment.

**Results:**

A total of 60 patients with 60 unruptured aneurysms were enrolled. Technical success rate was 98.3% (59/60). At 1 year, the treatment success rate was 54.2% (26/48), and two patients (3.9%) experienced an MAE, which was not device related. At 1 year, the complete occlusion rate was 56% and adequate occlusion rate was 82%. There were no retreatments, new bleeding events, or mortalities.

**Conclusions:**

This study demonstrated that the WEB device is safe and effective in the treatment of WNBAs in the Chinese population.

**Trial registration:**

Clinicaltrials.gov Unique Identifier NCT03207087.

**Supplementary Information:**

The online version contains supplementary material available at 10.1186/s41016-025-00418-2.

## Background

Intracranial aneurysms (IAs) affect 5–8% of the adult population worldwide [1]. In China, the prevalence of unruptured IAs is as high as 7% [2]. If left untreated, the consequence of aneurysm rupture can be catastrophic. For classic narrow-necked saccular aneurysms, conventional coiling is often sufficient; however, complex morphologies, such as wide-necked bifurcation aneurysms (WNBAs), still pose a serious treatment challenge and comprise 26–36% of all IAs [3]. Many coiling methods, including balloon-assisted, stent-assisted, and the waffle cone technique, have been used to treat WNBAs [4, 5]. However, coil embolization of WNBAs carries the risk of coil protrusion into the parent artery or coil migration in the distal circulation, resulting in thromboembolic complications. Use of flow diverters (FD) have been proposed to treat WNBAs, but efficacy has not been established, and FDs present unique safety issues, including the potential modification of bifurcation branches covered by the flow diverter [4]. Moreover, flow diversion requires the use of dual antiplatelet therapy (DAPT) [4].

Intrasaccular flow disruption devices are designed to be placed completely within the aneurysm sac, combining the benefits of flow diversion and disruption, as well as mechanical occlusion, while reducing the potential risks of DAPT. The Woven EndoBridge (WEB) device (TerumoNeuro, CA, USA) is the first intrasaccular flow disruption device on the market. Clinical studies have been conducted in Europe (WEB Clinical Assessment of Intrasaccular Aneurysm Therapy-WEBCAST and WEBCAST-2, French Observatory) and in the USA (WEB Intra-saccular Therapy (WEB-IT). All WEB studies have shown comparable positive results in terms of complete occlusion rate and low rates of morbidity and mortality, indicating the safety and efficacy of WEB in elective as well as acute cases [6–14]. However, the safety and efficacy of WEB have not been fully established in Chinese WNBA patients.

The WEB-IT China study was the first and only good clinical practice (GCP) study of intrasaccular flow disruption devices conducted in China, with the purpose of evaluating the safety and effectiveness of the WEB device for treating patients with WNBAs in the Chinese population.

## Methods

### Study design

**Fig. 1:**
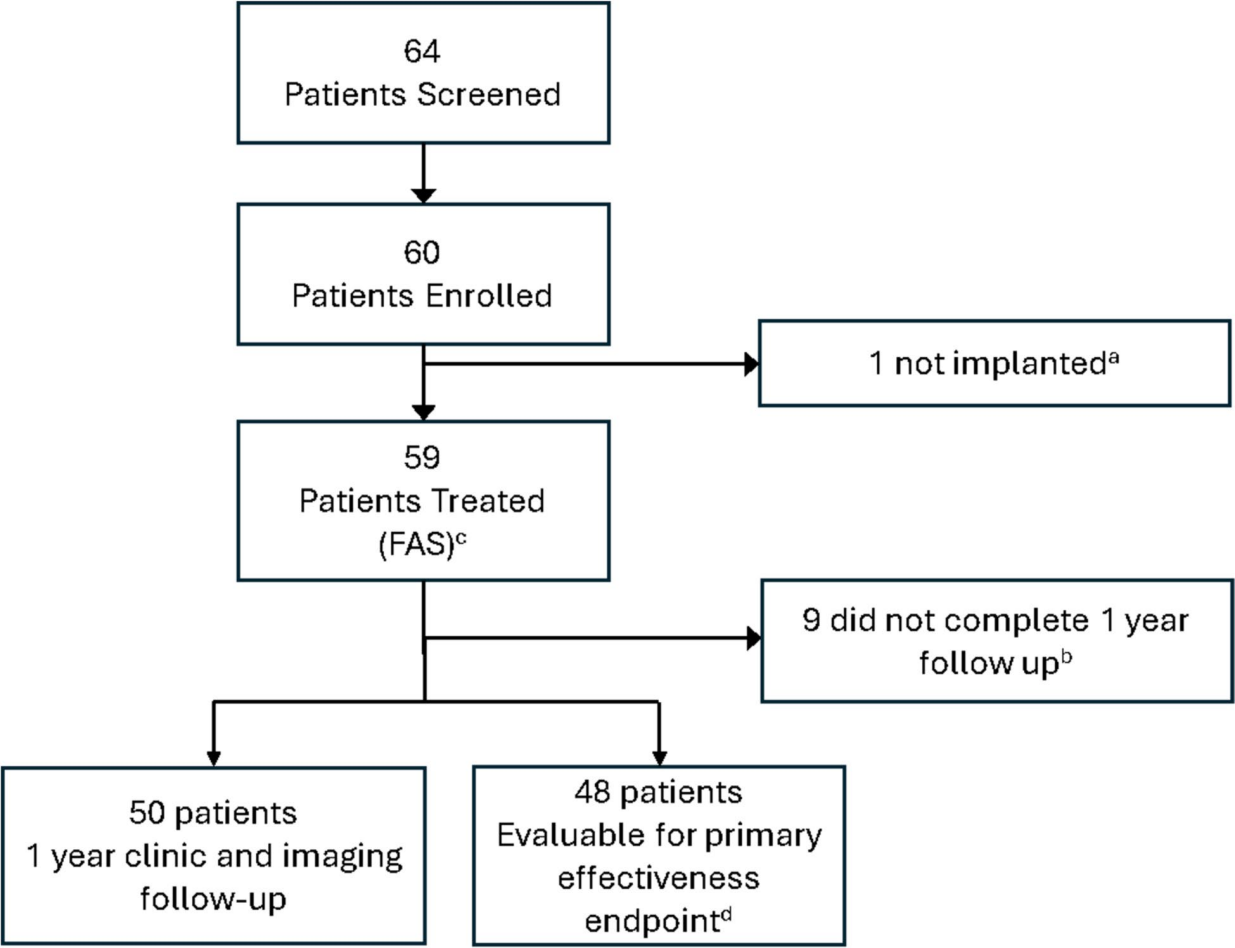
Patient disposition flowchart. **a** One patient was not implanted due to lack of appropriate device size. **b** Eight (8) patients were lost to follow up and one patient refused to return for 1 year visit. **c** Full analysis set (FAS) is defined as all enrolled patients that were implanted with the WEB device. **d** Patients that had evaluable imaging data for occlusion and stenosis

WEB-IT China was a prospective, single-arm confirmatory study conducted in 8 centers in China between June 2017 and August 2019. This study was conducted according to the requirements of the Declaration of Helsinki and Good Clinical Practice for Medical Devices (Decree 25 of China Food and Drug Administration and National Health and Family Planning Commission of the People’s Republic of China). The appropriate Ethics Committees reviewed and approved the informed consent for this study. An independent Core Laboratory adjudicated all angiographic outcomes, and an independent Clinical Events Committee (CEC) adjudicated all serious adverse events (SAEs) and safety endpoint events Fig. 1.

### Device characteristics

The WEB Aneurysm Embolization System (Terumo Neuro Inc., Aliso Viejo, California, USA) consists of an implantable embolization device attached to a delivery device. The delivery device is powered by a hand-held, battery-powered detachment control device designed specifically for the WEB Aneurysm Embolization System. The detachment control device is provided separately. The WEB embolization device is manufactured from nitinol wires in a braided, self-expanding mesh configuration [15, 16]. Manufactured under US Food and Drug Administration (FDA) and international quality system requirements (21 CFR Part 820) and International Organization for Standardization (ISO) 13485:2003. The embolization materials of the WEB Aneurysm Embolization System consist of WEB-SL and WEB-SLS. The characteristics of the WEB device have been detailed previously [11, 17]. WEB models and sizes used in this study were WEB-SL (diameter x height; mm) 4 × 3mm, 5 × 3mm, 6 × 3mm, 6 × 4mm, 6 × 5mm, 7 × 3mm, 7 × 4mm, 7 × 5mm, 8 × 3mm, 8 × 4mm, 8 × 5mm, 8 × 6mm, 9 × 4mm, 9 × 5mm, 9 × 6mm, 9 × 7mm, and 10 × 5 mm (mm) and WEB-SLS (diameter; mm) 6, 9, and 10 mm. All were the original WEB configuration, delivered through a VIA 21, 27, or 33 microcatheter.

### Procedures

All investigators participating in the study had prior experience with neuro-endovascular devices and underwent comprehensive training in the use of the WEB device, including structured sessions covering procedural overview and device handling, as well as hands-on experience with device deployment prior to enrolling patients.

Baseline data collected prior to the procedure included patient demographics, medical history, aneurysm characteristics (rupture status, location, and aneurysm size), physical and neurological exam, and mRS assessment. All aneurysms underwent pre-procedural digital subtraction angiography (DSA) to assess size, morphology, and parent artery anatomy and cases were reviewed and approved by an enrollment committee, which consisted of 5 physicians. Inclusion required saccular morphology, bifurcation location (AComA, MCA, ICAt, or BA apex), and wide-neck configuration (neck ≥ 4 mm or dome-to-neck ratio < 2, with an additional requirement of dome-to-neck ratio ≥ 1). Patients suitable for WEB treatment without the assistance of other implantable devices were enrolled.

The procedure was performed under general anesthesia. Antiplatelet and anticoagulant therapies were administered according to site-specific clinical protocols and appropriately documented. Initiation typically occurred prior to or on the day of the procedure. Peri-procedural anticoagulation with heparin was guided by institutional standards.

WEB sizing was determined based on aneurysm size and anatomy, and implantation was performed according to the standard interventional techniques. WEB devices were delivered using a VIA 21, VIA 27, or VIA 33 microcatheter (Terumo Neuro, Inc., Aliso Viejo, California, USA), depending on the WEB implant size selected for each procedure. All microcatheters were navigated and positioned with the aid of an appropriately sized guidewire.

Intraoperative imaging confirmed safe device navigation and parent vessel patency. Following WEB placement in the aneurysm, detachment was performed using a WEB Detachment Controller (Terumo Neuro, Inc., Aliso Viejo, California, USA). Additional required items to perform the procedure were a guide catheter compatible with the selected microcatheter, two rotating hemostatic Y valves (RHV), one three-way stopcock, one one-way stopcock, sterile saline, and a pressurized sterile saline drip. Patients were discharged after post-procedure recovery.

Procedural data collected included the size and model of the WEB used, perioperative antithrombotic medications, procedure time, and use of any additional devices during the procedure. Information on discharge status, including hospital stay, mRS, and the occurrence of any post-procedure AEs, was recorded.

Clinical follow-up was performed at 30 days, 6 months, and 1 year. Pharmacologic management was systematically documented peri-procedurally, and during follow-up at 30 days, 6 months, and 1 year. Any retreatments or retreatment plans for the target lesion were recorded at 6 months and 1 year. Imaging follow-ups were performed and assessed at 6 months and 12 months post-procedure, with 3D angiography preferred. All images were submitted for assessment to an independent core laboratory and graded per the modified Raymond Scale [18]. Safety data collected included adverse events (AEs), including post-procedure device- or procedure-related SAEs, safety endpoint events, device-related neurological complications, device- or procedure-related strokes, and new bleeding events.

### Study endpoints

The primary effectiveness endpoint was the treatment success rate at 1 year, which was defined as complete aneurysm occlusion without retreatment, recurrent SAH, or > 50% stenosis in the parent artery 1 year after treatment. A grading scheme consistent with the modified Raymond Scale was employed to assess the occlusion status in line with prior published aneurysm studies and as described in the US WEB-IT study and other scientific publications [11, 19, 20]. Parent artery stenosis was defined as a reduction in parent artery diameter just proximal or distal to the treated aneurysm from pre- to post-procedure imaging. The extent of parent artery stenosis was graded on a 3-point scale [20]: no reduction (0), ≤ 50% reduction (1), or > 50% reduction (2).

The primary safety endpoint was the proportion of patients with a major adverse event (MAE), a composite endpoint consisting of any non-accidental death or major stroke (≥ 4-point increase in NIHSS score) within 30 days of treatment or major ipsilateral stroke or neurological death from day 31 to 1 year after treatment [16, 21].

The secondary effectiveness endpoint was the proportion of patients with angiographic aneurysmal recurrence (defined as aneurysm growth or recanalization) at 1 year after treatment. Technical success was defined as the successful implantation of WEB during the procedure. Clinical outcome was assessed using mRS [22].

AE evaluation included assessment of severity, potential relationship to the study device and/or procedure dates of onset and resolution (if resolved), and immediate medications and/or treatment provided.

### Statistical analysis

This study planned to enroll 60 patients. The full analysis set (FAS) was determined according to the intention-to-treat (ITT) principle and consisted of all patients who participated in the study and used the investigational device. The analysis of primary safety and effectiveness endpoints, as well as baseline demographic data and secondary endpoints was based on the FAS. The general safety evaluation was also performed on the FAS.

Continuous data were expressed as mean ± standard deviations (SD) or median (range). Categorical variables were expressed as frequency (%). The 95% CI was calculated using the asymptotic normality method. All statistical analyses were conducted with a significance level of 0.05. Missing data for the primary effectiveness and safety endpoints were handled with the multiple imputation method, and the data set that was imputed 20 times was combined according to the Rubin Principles to estimate the primary effectiveness endpoint and 95% CI at 1 year. Statistical analyses were conducted using SAS® statistical software 9.4 (SAS Institute, Inc., Cary, NC, USA).

## Results

### Patient allocation and baseline characteristics

A total of 64 patients were screened, and 60 patients were enrolled in the study. One enrolled patient was not implanted with WEB due to appropriate device size not being available and was excluded from the analysis; thus, the FAS consisted of 59 patients.

Nine patients (15.3%) discontinued early; 8 were lost to follow-up and 1 patient refused to return for the 1 year follow-up visit. Overall, 50/59 (84.7%) patients completed the study, all had clinical and imaging follow up data available, but only 48 patients had both occlusion and stenosis readings rated by the core lab at 1 year follow-up.

Baseline characteristics for the FAS are presented in Table 1. The mean age was 61.0 (SD 9.1) years, and most patients were female (59.3%, 35/59). At baseline, 52/59 (88.1%) patients had an mRS score of 0, and 7/59 (11.9%) had an mRS score of 1. 49.2% (29/59) of the aneurysms were located in the AComA, 32.2% (19/59) in the MCA bifurcation, 16.9% (10/59) in the BA apex and 1.7% (1/59) in the ICA terminus. In this study population, intracranial aneurysms with dome-to-neck (DN) ≥ 1 and < 2 were included. The mean aneurysm height was 5.98 ± 1.79 mm (range: 2.09–10.4 mm) and the mean aneurysm width was 6.33 ± 1.86 mm (range: 3.03–10.56 mm), reflecting the overall aneurysm size distribution. The mean neck width was 4.71 ± 1.31 mm (range: 2.58–7.82 mm).

**Table 1:** Patient and aneurysm baseline characteristics

Characteristic	Frequency
Age (years)	61.0 ± 9.1 (*N* = 59)
Female sex	35/59 (59.3%)
Medical History	
Ischemic stroke	2/59 (3.4%)
Hemorrhagic stroke	3/59 (5.1%)
Dyskinesia	1/59 (1.7%)
Smoking history	8/59 (13.6%)
Baseline mRS	
0	52/59 (88.1%)
1	7/59 (11.9%)
Baseline NIHSS	
0	58/59 (98.3%)
1	1/59 (1.7%)
Aneurysm location	
BA apex	10/59 (16.9%)
MCA Bifurcation	19/59 (32.2%)
ICA terminus	1/59 (1.7%)
AComA	29/59 (49.2%)
Side	
Right	12/59 (20.3%)
Left	17/59 (28.8%)
Median line	30/59 (50.8%)
Rupture Status	
Unruptured	59/59 (100%)
Presentation	
Incidental finding	23/59 (39.0%)
Symptomatic	36/59 (61.0%)
Headache	15/36 (41.7%)
Other symptoms	22/36 (61.1%)
Aneurysm Dimensions, median (mean ± SD) (all enrolled patients)	
Aneurysm height (AP view), n (%)	5.90 (5.98 ± 1.79)
Aneurysm width (AP view), n (%)	6.15 (6.33 ± 1.86)
Aneurysm neck (AP view), n (%)	4.59 (4.71 ± 1.31)

Data presented as mean ± SD or n (%)

Data represents the full analysis set unless specified

*AComA* Anterior communicating artery, *AP* Anteroposterior, *BA* Basilar artery, *ICA* Internal carotid artery, *MCA* Middle cerebral artery, *mRS* modified Rankin Scale, *NIHSS* National Institutes of Health Stroke Scale, *SD* Standard deviation

### Procedural characteristics

Procedural characteristics of all enrolled patients are presented in Table 2. The WEB-SL model was used in 56/60 (93.3%) patients, and the WEB-SLS model was used in 4/60 (6.7%) patients. Coils and Flow Diverters (FD) were not used in any of the procedures. Balloons were used in 2/60 (3.3%) patients. In both cases, the procedure was completed successfully without any complications.

**Table 2:** Procedural characteristics of all enrolled patients

Characteristic	Frequency (*N* = 60)
Vascular puncture site	
Left femoral artery only	4 (6.7%)
Right femoral artery only	56 (93.3%)
Successful implantation of the WEB device during the procedure	59 (98.3%)
WEB model used	
SL model^a^	56 (93.3%)
SLS model	4 (6.7%)
Procedure time, min	17.59 ± 13.30 (*N* = 59)
Use of an ancillary device during WEB procedure	5 (8.3%)
Embolization coil used	0 (0.0%)
Balloon used	2 (3.3%)
Flow diverter used	0 (0.0%)
Use of antiplatelet or anticoagulant drugs	46 (76.7%)

Data presented as mean ± SD or n (%)

*WEB* Woven EndoBridge

^a^ Includes one device that was not implanted due to improper size

The mean procedure time was 17.59 ± 13.30 min. Intra-procedural antiplatelet or anticoagulant therapy was administered in 46/59 (77.9%) of patients. Antiplatelet or anticoagulant were administered in 38/59 (64.4%) patients at discharge, 40/59 (67.8%) at 30 days, 26/59 (45.6%) at 6 months, and 11/59 (22%) at 1 year. Further data are presented in Table 3.

**Table 3:** Summary of intraoperative and postoperative antiplatelet and anticoagulant therapy

Timepoint	Medication / Regimen	No. of Patients (%)
Intraoperative	Any antiplatelet and/or anticoagulant therapy	46/59 (77.9%)
At discharge		38/59 (64.4%)
30-day follow-up		40/59 (67.8%)
6-month follow-up		26/59 (45.6%)
1 year follow-up		11/50 (22.0%)

### Primary effectiveness endpoint

Primary effectiveness endpoint results are presented in Table 3. At 1 year, data were available for 48/59 (81.4%) patients in the FAS, out of which 26/48 (54.2%, 95% CI 39.0–69.3%) patients met the primary effectiveness endpoint. Multiple imputation for missing data yielded an estimated success rate of 54.5% (95% CI 39.9–69.1%).

### Secondary effectiveness endpoint

Secondary effectiveness endpoint results were determined based on the FAS (Table 4). Of the 50/59 (84.7%) patients that had evaluable 1 year angiographic follow-up, 7/50 (14.0%) had aneurysm recurrence.

**Table 4:** Effectiveness and clinical outcomes

Outcome	n/N (%)
Primary effectiveness endpoint (Core Laboratory)	
1 year	26/48 (54.2%)
	95% CI (39.0%−69.3%)
1 year (imputation method)	54.5% 95% CI (39.9%−69.1%)
Secondary effectiveness endpoint (Core Laboratory)	
Aneurysm recurrence at 1 year follow-up	7/50 (14.0%)
Aneurysm occlusion status at 6 months (Core Laboratory)	
Complete occlusion	28/57 (49.1%)
Residual aneurysm neck	16/57 (28.1%)
Residual aneurysm	13/57 (22.8%)
Aneurysm occlusion status at 1 year (Core Laboratory)	
Complete occlusion	28/50 (56.0%)
Residual aneurysm neck	13/50 (26.0%)
Residual aneurysm	9/50 (18.0%)
Aneurysm occlusion stability (6 month to 1 year) (Core Laboratory)	
Identical	40/49 (81.6%)
Improved	6/49 (12.2%)
Worsened	3/49 (6.1%)
Retreatments	
6 months	0/57 (0.0%)
1 year	0/50 (0.0%)
Clinical outcome	
mRS 0–2 at 30 days	58/59 (98.3%)
mRS 0–2 at 6 months	56/56 (100.0%)
mRS 0–2 at 1 year	49/49 (100.0%)
Angiographic outcome and modality	
6 months	
3D angiography	57/57 (100.00%)
1 year	
3D angiography	49/50 (98.0%)
Magnetic Resonance Imaging	1/50 (2.0%)

Data represents the full analysis set unless specified

*mRS* modified Rankin Scale

### Aneurysm occlusion and occlusion stability

From the FAS dataset, angiographic follow-up was available for 57/59 (96.6%) patients at 6 months and 50/59 (84.7%) at 1 year. At 6 months, complete aneurysm occlusion was achieved in 28/57 (49.1%) patients, with residual neck in 16/57 (28.1%) and residual aneurysm in 13/57 (22.8%). At 1 year, complete occlusion was seen in 28/50 (56.0%) patients, with residual neck in 13/50 (26.0%), and residual aneurysm in 9/50 (18.0%).

Occlusion stability data were available for 49 patients. compared to 6 months, 1 year occlusion status was identical in 40/49 (81.6%) patients, improved in 6/49 (12.2%), and worsened in 3/49 (6.1%). No patients underwent or had planned retreatment of the target lesion at 6 months or 1 year.

### Clinical outcomes

Other endpoint results are presented in Table 4. The rate of mRS 0–2 at 30 days was 58/59 (98.3%), at 6 months was 56/56 (100%), and at 1 year was 49/49 (100%). At discharge and all subsequent follow-up visits, most patients' mRS scores were unchanged, with a score of 0 at baseline and at each follow-up. Modified Rankin score of 0 was reported in 45/59 (76.3%) of patients at discharge, 47/59 (79.7%) of patients at 30 days, 43/56 (76.8%) of patients at 6 months, and 41/49 (83.7%) of patients at 1 year.

### Primary safety endpoint

Primary safety endpoint results are presented in Table 5. In the FAS at 1 year, the MAE rate based on the multiple imputation method to account for missing patients was 3.8% (FAS).

**Table 5:** Safety outcomes

Outcome	n/N (%)
Primary safety endpoint (CEC)	
MAEs at 1 year after treatment (FAS)*	2/51 (3.9%) 95% CI (0.0%−10.2%)
MAEs at 1 year after treatment (imputation method) (FAS)	3.8% 95% CI (0.0%−9.0%)
Other safety outcomes (FAS)	
AEs within 1 year (site reported)	27/59 (45.8%) [*n* = 46]
SAEs within 1 year (site reported)	12/59 (20.3%) [*n* = 13]
Device-related AEs at 1 year (CEC)	1/59 (1.7%)
Device-related SAEs at 1 year (CEC)	0/59 (0.0%)
Intraoperative device-related neurological complications (CEC)	0/59 (0.0%)
Device- and procedure-related stroke at 30 days (CEC)	3/59 (5.1%)
Device- and procedure-related death at 30 days (CEC)	0/59 (0.0%)
Mortality at 1 year (CEC)	0/50 (0.0%)
New bleeding events for target unruptured aneurysms at 1 year (Site reported)	0/50 (0.0%)

Data represents the full analysis set (FAS) unless specified

*AE* Adverse event, *CEC* Clinical Events Committee, *MAE* Major adverse event, *SAE* Serious adverse event, *EDC* Electronic data capture

^*^Major adverse events at the 1 year of follow-up included any non-accidental death or major stroke (postoperative NIHSS score increased by ≥ 4 points) within 30 days after treatment or major ipsilateral stroke or death was definitively caused by neurological etiology occurring from 31 days to 1 year after treatment

The two reported events were adjudicated by the CEC as major strokes possibly related to an underlying disease or concurrent condition and unrelated to the WEB device or procedure. One patient experienced left thalamus and basal ganglia bleeding about 2 h after the procedure, and the other patient experienced acute cerebral infarction (left basal ganglia) shortly after the 6 month follow-up. Both patients had a history of hypertension.

### Other safety outcomes

Other safety outcomes for the FAS are presented in Table 5. In the FAS, a total of 46 site reported AEs occurred in 27/59 (45.8%) patients, and 13 site reported SAEs occurred in 12/59 (20.3%) patients. Postoperative device and procedure related AEs were reported in a total of 5 patients (8.5%). Per CEC, three patients (5.1%) experienced device and procedure related stroke, all of which were minor and occurred within 1 day post procedure. Two of these events were procedure related and 1 event was device related. Of note, this is the only device related adverse event that occurred in the study. There were no deaths and no bleeding events in patients with WEB treated aneurysms.

## Discussion

The WEB-IT CHINA study demonstrated the safety and effectiveness of WEB for the treatment of WNBAs in a Chinese population, with 54.2% meeting the primary effectiveness endpoint and 3.9% experiencing MAEs at 1 year.

This study showed a 56% rate of complete occlusion and an 82% rate of adequate occlusion at 1 year, with 93.8% showing stable or progressive occlusion between 6 months and 1 year.

There were no retreatments performed up to 1 year post procedure. The data revealed an excellent safety profile as demonstrated by no mortalities, device related SAEs, or new bleeding events [23]. Additionally, there was no morbidity (defined as mRS ≥ 3) at 1 year follow-up.

While the 1 year complete occlusion rate in this study was not as high as that reported for sidewall aneurysms, it is well known that the anatomy of WNBAs poses unique challenges for treatment, making them among the most difficult saccular aneurysms to treat [24, 25]. WNBAs are usually associated with a high risk of recanalization, branch occlusion, coil protrusion, and thromboembolic complications when treated with coiling, and the complete occlusion rate in coiled wide neck aneurysms is significantly lower than that reported for all unruptured aneurysms (27.1% vs. 44.4%) at 1 year follow-up. Even with treatment using stent assisted coiling (SAC), the complete occlusion rate in wide neck aneurysms is also significantly lower than that reported for all unruptured aneurysms (45.7% vs 51.8%) at 1 year follow-up. It is likely that WNBAs would have yielded even lower rates of complete occlusion [26]. Additionally, our study enrolled a high proportion of AcomA (49.2%) and MCA (32.2%) WNBAs, which are notoriously difficult to treat due to their complex structure and proximity to critical vessels. The high percentage of AcomA and MCA WNBAs in our study could result in a reduced rate of complete occlusion. Interestingly, despite the higher proportion of AComA aneurysms, the effectiveness and safety outcomes in WEB-IT China were comparable to those reported in WEB-IT US, and the cumulative results of 3 GCP studies in Europe [6, 11]. However, WEB-IT China did not enroll patients with ruptured aneurysms, while WEB-IT US enrolled 6% of patients with ruptured aneurysms [11].

This study demonstrated an increased rate of complete occlusion over time and the absence of rupture/rebleeding, indicating the durability of the WEB treatment over time. These outcomes are even more promising when compared to the existing non-WEB treatment data for WNBAs, showing relatively low rates of complete occlusion (~ 45%) and adequate occlusion (~ 60%) in addition to relatively high rates of complications as assessed by safety endpoints (~ 19%).

Notably, the complication rates associated with WEB were very low in our study. Stroke occurred in 3/59 (5.1%) within 30 days, with the CEC adjudicating 2 as procedure-related and 1 as device-related. All 3 strokes were minor and non-disabling.

Also, no morbidity, mortalities, new bleeding events, or device-related SAEs were found at 1 year. Another study reported 2% bleeding rate in a systematic review of WEB for ruptured and unruptured WNBAs. Progressive technological improvements in the design of the WEB have helped decrease thromboembolic complications, and the outcomes could continue to improve with better deployment of the device and better patient selection [27, 28]. Furthermore, there were no cases where a flow diverter or coils were used and balloons were rarely required.

This study expands on the WEB-IT U.S. trial, extending the findings to the Chinese population. WEB-IT China represents a GCP-compliant multicenter evaluation conducted outside of Western countries reporting the outcomes of WNBA treatment with the WEB device. WEB-IT China had a similar study design to the U.S. WEB-IT study, with a smaller population, single-arm design, and the same primary effectiveness and safety endpoints. Complete and adequate occlusion rates at 1 year period were comparable (53.8% vs 56%, 84.6% vs 82%), with an overall low rate of primary safety events (0.7% vs. 3.9%). The recurrence rates are similar (11% vs 14%); however, the retreatment rates are different (5.6% vs 0) at 1 year follow-up. This can potentially be explained by operator and regional differences in retreatment paradigms, as no criteria for retreatment were specified in the protocol. Furthermore, the nuanced cultural, societal, and genetic contexts that are unique to the Chinese population could potentially influence the outcome and interpretation of WEB interventions. Collectively, these studies provide consistent data regarding the efficacy and safety of WEB in WNBAs across geographies.

There are several limitations to our study. First, this was a single-arm non-randomized study that lacked a control group. Thus, head-to-head effectiveness and safety comparison of WEB and other existing treatments for WNBAs are lacking. Second, while the study protocol allowed both ruptured and unruptured patients to enroll, the enrolled population included patients with unruptured aneurysms only. Thus, the generalizability of the results to the real-world patient population beyond those with unruptured aneurysms is limited. Third, DAPT management was not uniform. Fourth, the study population represented a small sample size. Finally, longer term follow-up is needed to assess longer term angiographic results.

## Conclusion

This study demonstrated the safety and effectiveness of the WEB device in treating WNBAs in a Chinese population, with high rates of complete occlusion and occlusion stability. There were no retreatments, mortalities, or new bleeding events up to 1 year after treatment, demonstrating an excellent safety profile. Moreover, the outcomes reported in this study were comparable to GCP studies conducted in Europe and the U.S. These findings further support the safety and effectiveness of the WEB device in the treatment of intracranial WNBAs and provide early confirmation of the generalizability of results across populations and geographies.

## Supplementary Information

Supplementary Material 1.

## Data Availability

Data will be made available upon reasonable request by the corresponding author **.**

## Abbreviations

AcomA: Anterior communicating artery
AEs: Adverse Events
BA: Basilar Apex
CEC: Clinical Events Committee
DAPT: Dual antiplatelet therapy
DFT: Drawn filled tube
FAS: Full Analysis Set
GCP: Good Clinical Practice
ICAt: Internal Carotid Artery Terminus
ITT: Intention to Treat
MAE: Major Adverse Event
MCA: Middle Cerebral Artery
PP: Per Protocol
SAH: Subarachnoid Hemorrhage
SAEs: Serious Adverse Events
SD: Standard Deviation
WEB: Woven EndoBridge
WEBCAST: WEB Clinical Assessment of Intrasaccular Aneurysm Therapy
WEB-IT: WEB Intra-saccular Therapy
WNBAs: Wide-necked bifurcation aneurysms

## References

[1] Rinkel GJ, Djibuti M, Algra A, van Gijn J. Prevalence and risk of rupture of intracranial aneurysms: a systematic review. Stroke. 1998;29(1):251–6. 9445359 10.1161/01.str.29.1.251

[2] Zheng E, Xu J, Xu J, Zeng X, Tan WJ, Li J, et al. Health-related quality of life and its influencing factors for elderly patients with hypertension: evidence from Heilongjiang Province, China. Front Public Health. 2021;9:654822. 33796501 10.3389/fpubh.2021.654822 PMC8007785

[3] Goyal N, Hoit D, DiNitto J, Elijovich L, Fiorella D, Pierot L, et al. How to WEB: a practical review of methodology for the use of the Woven EndoBridge. J Neurointerv Surg. 2020;12(5):512–20. 32005760 10.1136/neurintsurg-2019-015506 PMC7231463

[4] Pierot L, Biondi A. Endovascular techniques for the management of wide-neck intracranial bifurcation aneurysms: a critical review of the literature. J Neuroradiol. 2016;43(3):167–75. 26976346 10.1016/j.neurad.2016.02.001

[5] Goertz L, Liebig T, Siebert E, Pflaeging M, Forbrig R, Pennig L, et al. Stent-assisted WEB embolization: aneurysm characteristics, outcome and case report of a WEB delivered through a stent. Acta Neurochir. 2022;164:2181–90. 35037115 10.1007/s00701-022-05115-y PMC9337996

[6] Pierot L, Moret J, Barreau X, Szikora I, Herbreteau D, Turjman F, et al. Safety and efficacy of aneurysm treatment with WEB in the cumulative population of three prospective, multicenter series. J Neurointerv Surg. 2018;10(6):553–9. 28965106 10.1136/neurintsurg-2017-013448 PMC5969386

[7] Pierot L, Costalat V, Moret J, Szikora I, Klisch J, Herbreteau D, et al. Safety and efficacy of aneurysm treatment with WEB: results of the webcast study. J Neurosurg. 2016;124(5):1250–6. 26381253 10.3171/2015.2.JNS142634

[8] Pierot L, Gubucz I, Buhk JH, Holtmannspötter M, Herbreteau D, Stockx L, et al. Safety and efficacy of aneurysm treatment with the WEB: results of the WEBCAST 2 study. Am J Neuroradiol. 2017;38(6):1151–5. 28450432 10.3174/ajnr.A5178 PMC7960101

[9] Pierot L, Moret J, Turjman F, Herbreteau D, Raoult H, Barreau X, et al. WEB treatment of intracranial aneurysms: clinical and anatomic results in the French observatory. Am J Neuroradiol. 2016;37(4):655–9. 26514608 10.3174/ajnr.A4578 PMC7960156

[10] Pierot L, Moret J, Turjman F, Herbreteau D, Raoult H, Barreau X, et al. WEB treatment of intracranial aneurysms: feasibility, complications, and 1-month safety results with the WEB DL and WEB SL/SLS in the French observatory. Am J Neuroradiol. 2015;36(5):922–7. 25655876 10.3174/ajnr.A4230 PMC7990613

[11] Arthur AS, Molyneux A, Coon AL, Saatci I, Szikora I, Baltacioglu F, et al. The safety and effectiveness of the Woven EndoBridge (WEB) system for the treatment of wide-necked bifurcation aneurysms: final 12-month results of the pivotal WEB Intrasaccular Therapy (WEB-IT) Study. J Neurointerv Surg. 2019;11(9):924–30. 30992395 10.1136/neurintsurg-2019-014815 PMC6824604

[12] Ten PL. Years of clinical evaluation of the Woven EndoBridge: a safe and effective treatment for wide-neck bifurcation aneurysms. Neurointervention. 2021;16(3):211–21. 34674453 10.5469/neuroint.2021.00395 PMC8561039

[13] Monteiro, A, Lazar, AL, Waqas, M, Rai, HH, Baig, AA, Cortez, GM, Dossani, RH, Cappuzzo, JM, Levy, EI, Siddiqui, AH. Treatment of ruptured intracranial aneurysms with the Woven EndoBridge device: a systematic review. J Neurointerventional Surg. 2022;14:366-370. 10.1136/neurintsurg-2021-017613 34266907

[14] Aguiar, G, Caroff, J, Mihalea, C, Cortese, J, Girot, JB, Elawady, A, Vergara Martinez, J, Ikka, L, Gallas, S, Chalumeau, V, Ozanne, A, Moret, J, Spelle, L. WEB device for treatment of posterior communicating artery aneurysms. J Neurointerventional Surg. 2022;14:362-365. 10.1136/neurintsurg-2021-017405 33975921

[15] Ding YH, Lewis DA, Kadirvel R, Dai D, Kallmes DF. The woven endobridge: a new aneurysm occlusion device. Am J Neuroradiol. 2011;32(3):607–11. 21330397 10.3174/ajnr.A2399 PMC8013102

[16] Brott T, Adams HP Jr, Olinger CP, Marler JR, Barsan WG, Biller J, Spilker J, Holleran R, Eberle R, Hertzberg V, et al. Measurements of acute cerebral infarction: a clinical examination scale. Stroke. 1989;20(7):864-70. 10.1161/01.str.20.7.864. PMID: 2749846. 10.1161/01.str.20.7.864 2749846

[17] Gajera, J, Maingard, J, Foo, M, Ren, Y, Lamanna, A, Nour, D, Hall, J, Kurda, D, Tan, D, Lalloo, S, Bañez, RMF, Russell, J, Slater, LA, Chandra, RV, Chong, W, Jhamb, A, Brooks, DM, Asadi, H. The Woven EndoBridge device for the treatment of intracranial aneurysms: initial clinical experience within an Australian population. Neurointervention. 2022. 17:28-36. 10.5469/neuroint.2021.00430 PMC8891585 35130672

[18] Roy D, Milot G, Raymond J. Endovascular treatment of unruptured aneurysms. Stroke. 2001;32(9):1998–2004. 11546888 10.1161/hs0901.095600

[19] Fiorella D, Arthur A, Byrne J, Pierot L, Molyneux A, Duckwiler G, et al. Interobserver variability in the assessment of aneurysm occlusion with the WEB aneurysm embolization system. J Neurointerv Surg. 2015;7(8):591–5. 24984707 10.1136/neurintsurg-2014-011251

[20] Bahar A, Kohar RC, Gunawan A, et al. Single versus multiple coverage of pipeline embolization device for treatment of intracranial aneurysms: a systematic review. Egypt J Neurol Psychiatry Neurosurg. 2023;59:130. 10.1186/s41983-023-00713-8.

[21] Fiorella D, Molyneux A, Coon A, Szikora I, Saatci I, Baltacioglu F, et al. Safety and effectiveness of the Woven EndoBridge (WEB) system for the treatment of wide necked bifurcation aneurysms: final 5 year results of the pivotal WEB Intra-saccular Therapy study (WEB-IT). J Neurointerv Surg. 2023;15:1175–80. 37355252 10.1136/jnis-2023-020611 PMC10715507

[22] Saver JL, Chaisinanunkul N, Campbell BCV, Grotta JC, Hill MD, Khatri P, et al. Standardized nomenclature for modified rankin scale global disability outcomes: consensus recommendations from stroke therapy academic industry roundtable XI. Stroke. 2021;52(9):3054–62. 34320814 10.1161/STROKEAHA.121.034480

[23] Fiorella D, Molyneux A, Coon A, Szikora I, Saatci I, Baltacioglu F, et al. Demographic, procedural and 30-day safety results from the WEB Intra-saccular Therapy Study (WEB-IT). J Neurointerv Surg. 2017;9(12):1191–6. 28096478 10.1136/neurintsurg-2016-012841

[24] Trivelato FP, Salles Rezende MT, Ulhôa AC, Henrique de Castro-Afonso L, Nakiri GS, Abud DG. Occlusion rates of intracranial aneurysms treated with the Pipeline embolization device: the role of branches arising from the sac. J Neurosurg. 2018;130:1–7. 10.3171/2017.10.JNS172175 29624153

[25] Fiorella D, Arthur AS, Chiacchierini R, Emery E, Molyneux A, Pierot L. How safe and effective are existing treatments for wide-necked bifurcation aneurysms? Literature-based objective performance criteria for safety and effectiveness. J Neurointerv Surg. 2017;9(12):1197–201. 28798268 10.1136/neurintsurg-2017-013223

[26] Hetts SW, Turk A, English JD, Dowd CF, Mocco J, Prestigiacomo C, et al. Stent-assisted coiling versus coiling alone in unruptured intracranial aneurysms in the matrix and platinum science trial: safety, efficacy, and mid-term outcomes. Am J Neuroradiol. 2014;35(4):698–705. 24184523 10.3174/ajnr.A3755 PMC7965822

[27] Lv X, Zhang Y, Jiang W. Systematic review of woven endobridge for wide-necked bifurcation aneurysms: complications, adequate occlusion rate, morbidity, and mortality. World Neurosurg. 2018;110:20–5. 29107726 10.1016/j.wneu.2017.10.113

[28] Sabuzi F, Cortese J, Da Ros V, Mihalea C, Chalumeau V, Moret J, et al. How a decade of aneurysms embolization with the Woven EndoBridge has changed our understanding and practices? J Neuroradiol = J Neuroradiol. 2023;50:518–22. 10.1016/j.neurad.2023.02.006 36868371

