# Supplementary material for: Woven EndoBridge intrasaccular therapy for the treatment of unruptured wide-necked bifurcation aneurysms: a prospective study in a Chinese population
Source: Chin Neurosurg J. 2026 Jan 20;12:2. doi: 10.1186/s41016-025-00418-2 (PMC12817425; doi:10.1186/s41016-025-00418-2)
Supplement: Supplementary file 1 — Supplementary Material 1. [file 41016_2025_418_MOESM1_ESM.docx]

**SUPPLEMENTARY MATERIAL FOR WEB-IT CHINA STUDY**

**Woven EndoBridge Intrasaccular therapy for the treatment of unruptured wide-necked bifurcation aneurysms: a prospective study in Chinese population**

**Supplementary Table 1: Study inclusion and exclusion criteria**

| **Inclusion Criteria** | **Exclusion Criteria** |
| --- | --- |
| 1. Patient was aged 18-75 years at the time of screening. 2. Patient must have a single ruptured or unruptured IA requiring treatment. If the patient has an additional IA requiring treatment, the additional IA must not require treatment within 60 days of the index procedure.   Note: SAH as indicated by computed tomography (CT), magnetic resonance imaging (MRI), or lumbar puncture (LP) attributed to the index aneurysm within the last 60 days.   1. The index IA to be treated must have the following characteristics: 2. Saccular in shape 3. Located in the BA, MCA bifurcation, ICAt, or ACom 4. DN ratio ≥1 5. Diameter of the aneurysm meets the requirements in the IFU of the WEB Aneurysm Embolization System 6. Wide-neck IA with neck size ≥4 mm or DN ratio <2 7. The index aneurysm is suitable for WEB treatment without the assistance of other implantable devices. 8. If the IA previously ruptured, the patient must be neurologically stable, with Hunt-Hess score of I or II. 9. Patient must abide by all requirements of the screening, evaluation, treatment, and the post-procedure visits. 10. Patient must sign and date an EC-approved written informed consent before initiating any study procedures. | 1. Patient has an IA with characteristics unsuitable for endovascular treatment. 2. Microcatheter could not reach the patient’s IA, leading to treatment failure using the investigational device. 3. Patient has vessel characteristics, tortuosity, or morphology that could preclude safe access and support during treatment with the investigational device. 4. Patient has vascular disease or other vascular anomaly that precludes the necessary access to the aneurysm for use of the investigational device. 5. Patient has clinical, angiographic, or CT evidence of vasospasm, vasculitis, or intracranial tumor (except cerebellar meningioma) or any other intracranial vascular malformations. 6. Patient has conditions placing them at high risk for ischemic stroke or has exhibited ischemic symptoms such as transient ischemic attacks (TIAs), minor strokes, or progressive stroke within the past 60 days. 7. Patient has any circulatory, neurovascular, cardiovascular, or neurologic conditions that have resulted in unstable neurological symptoms. 8. Patient has a modified Rankin Scale (mRS) score ≥2 before presentation or rupture (as applicable). 9. Patient has had an SAH from a non-index IA or any other intracranial hemorrhage within 90 days. 10. Patient has physical, neurologic, or psychiatric conditions that preclude his/her ability to comply with all aspects of screening, evaluation, treatment, and post-procedure follow-up schedule. 11. Patient’s index IA was previously treated. 12. Patient is taking anticoagulants or has a known blood dyscrasia, coagulopathy, or hemoglobinopathy. 13. Patient is pregnant. 14. Patient has known hypersensitivity, which cannot be medically treated, to any component of the study device, procedural materials, or medications commonly used during the procedure. 15. Patient is concurrently involved in another investigational study or a post-market study that could affect the safety and effectiveness of IA treatment with the study device or with the study’s follow-up schedule. 16. Patient has an acute life-threatening illness other than the neurological disease being treated in the study. 17. Patient has a life expectancy <2 years due to other illness or condition (in addition to an IA). 18. Patient has a mental disorder. |

AComA=anterior communicating artery; BA=basilar artery; CT=computed tomography; DN=dome-to-neck; IA=intracranial aneurysm; ICA=internal carotid artery; LP=lumbar puncture; MCA=middle cerebral artery; MRI=magnetic resonance imaging; SAH=subarachnoid hemorrhage; TIA=transient ischemic attack; WEB=Woven EndoBridge.

**Supplementary Table 2:** **Schedule of assessments**

| **Parameter** | **Screening** | **Index procedure** | **Discharge from hospital** | **30-day follow-up** | **6-month follow-up** | **1-year follow-up** |
| --- | --- | --- | --- | --- | --- | --- |
| Medical history | X |  |  |  |  |  |
| Physical examination | X | X | X |  | X | X |
| Neurological  examination | X |  |  |  | X | X |
| Site/patient information | X |  |  |  |  |  |
| Aneurysm information  (eg, size, location) | X | X |  |  |  |  |
| Rupture status | X |  |  |  |  |  |
| Hunt and Hess grade  (ruptured aneurysms only) | X |  |  |  |  |  |
| Microcatheter(s) used |  | X |  |  |  |  |
| Ancillary devices used (eg, stent, balloon) |  | X |  |  |  |  |
| Medications used | X | X | X |  | X | X |
| WEB device(s) used (size, Lot Number) |  | X |  |  |  |  |
| WEB procedure and x-ray fluoroscopy |  | X |  |  |  |  |
| Total time of procedure and x- ray fluoroscopy |  | X |  |  |  |  |
| 3-D angiography | X | X |  |  | X | X |
| Liver/kidney function tests | X |  | X |  |  |  |
| Urine pregnancy test | X |  |  |  |  |  |
| Aneurysm occlusion assessment  (Core Laboratory) |  | X |  |  | X | X |
| Modified Rankin Scale | X |  | X | X | X | X |
| NIHSS score | X | As required* | | | | |
| QOL assessment (EQ-5D) |  |  |  |  | X |  |
| Other proper measuring scale |  | X |  |  | X | X |
| Technique related |  | X |  |  |  |  |
| Adverse events |  | X | X | X | X | X |
| Retreatments/additional  procedures |  |  |  | X | X | X |
| Recurrent bleeding (if  ruptured)/new bleeds |  |  | X | X | X | X |
| *NIHSS was assessed at baseline in all patients who experienced a suspected stroke within 7 days (range: 1–10 d) after ictus. | | | | | | |

NIHSS=National Institutes of Health Stroke Scale; QOL= Quality of Life; WEB=Woven EndoBridge

**Supplementary Table 3: Definitions for relationship of AE to study-related factors**

|  | **Unrelated** | **Unlikely related** | **Possibly related** | **Probably related** | **Positively related** |
| --- | --- | --- | --- | --- | --- |
| Study device | AE is related to another device, drug, or disease, and is not related to the study device. | While the AE could be related to the study device, an alternative explanation is more likely. | AE may be related to the study device, but it may also be caused by other factors. | Causal/temporal relationship to the study device is likely or significantly more likely than other plausible explanations. | AE can only be attributed to the study device. |
| Ancillary device | AE is not related to the use of an ancillary device. | While the AE could be related to the ancillary device, an alternative explanation is more likely. | AE may be related to the ancillary device, but it may also be caused by other factors. | Causal/temporal relationship to the ancillary device is likely or significantly more likely than other plausible explanations. | AE can only be attributed to the ancillary device. |
| Endovascular procedure | AE is not related to the procedure. | While the AE could be related to the procedure, an alternative explanation is more likely. | AE may be related to the procedure, but it may also be caused by other factors. | Causal/temporal relationship to the procedure is likely or significantly more likely than other plausible explanations. | AE can only be attributed to the procedure. |
| Study disease | AE is not related to worsening of the study disease. | While the AE could be related to the study disease, an alternative explanation is more likely. | AE may be related to the study disease, but it may also be caused by other factors. | Causal/temporal relationship to the study disease is likely or significantly more likely than other plausible explanations. | AE can only be attributed to the study disease. |
| Comorbid condition | AE is not related to the comorbid condition. | While the AE could be related to the comorbid condition, an alternative explanation is more likely. | AE may be related to the comorbid condition, but it may also be caused by other factors. | Causal/temporal relationship to the comorbid condition is likely or significantly more likely than other plausible explanations. | AE can only be attributed to the comorbid condition. |
